# Examining the Dynamic Association of BMI and Mortality in the Framingham Heart Study

**DOI:** 10.3390/ijerph6123115

**Published:** 2009-12-07

**Authors:** Jianghua He, Daniel McGee, Xufeng Niu, Won Choi

**Affiliations:** 1 Department of Biostatistics,University of Kansas Medical Center, Mail Stop 1026, 3901 Rainbow Blvd, Kansas City, KS, 66160, USA; 2 Department of Statistics, Florida State University,117 N. Woodward Ave. P.O. Box 3064330, Tallahassee, FL, 32306-4330, USA; E-Mails: dan@stat.fsu.edu (D.M.); niu@stat.fsu.edu (X.F.N.); 3 Department of Preventive Medicine and Public Health, University of Kansas Medical Center, Mail Stop 1008, 3901 Rainbow Blvd, Kansas City, KS, 66160, USA; E-Mail: wchoi@kumc.edu (W.C.)

**Keywords:** body mass index, mortality, time-varying association, dynamic survival models

## Abstract

Based on the 40-year follow-up of the Framingham Heart Study (FHS), we used logistic regression models to demonstrate that different designs of an observational study may lead to different results about the association between BMI and all-cause mortality. We also used dynamic survival models to capture the time-varying relationships between BMI and mortality in FHS. The results consistently show that the association between BMI and mortality is dynamic, especially for men. Our analysis suggests that the dynamic property may explain part of the heterogeneity observed in the literature about the association of BMI and mortality.

## 1. Introduction

Obesity is a major health concern in the United States and in the world. More and more evidence shows that it is related to a high incidence of diseases such as coronary heart disease (CHD), diabetes, and cancer, and mortality from those diseases. However, there appears to be no consensus on the relationship between body weight and mortality in epidemiological studies. For example, conclusions about the relationship between BMI and all-cause mortality include no association [1–3], a U-shaped [4, 5] or a J-shaped [1–3, 6–8] relation, a direct association (higher BMI related to higher risk) [9–11], and even an inverse association [12].

Several factors make it difficult to determine the true relationship between BMI and mortality using traditional observational studies and analysis methods. These factors include: (1) BMI often changes during a person’s lifetime but in most studies only BMI at baseline (the beginning of the follow-up period) is used for analysis; (2) a longer follow-up period covers more long-term effects and the length of follow-up may vary from several years to several decades; (3) analytic methods vary but have included cross-tabulations, logistic regression models, and the Cox survival models. Each method gives one or several numerical values (odds ratio, relative risk, or hazard ratio) summarizing the relationship between BMI (measured at a particular time point: baseline) and mortality (within a particular follow-up period).

**Figure 1.**
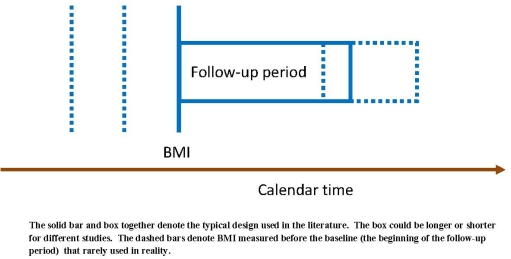
The design of an observational study about BMI and mortality.

The design of a study about BMI and mortality can be illustrated with Figure 1. The solid bar and box together denote the typical design used in the literature, where the bar is BMI at baseline and the box is the follow-up period. A study can be done differently, for example, using BMI at sometime before the baseline as denoted by the dashed bars in Figure 1, or following the population for a shorter or longer period of time. If there is a fixed association between BMI (at any time point) and mortality (within any period), different designs will lead to the same result, otherwise, different designs will naturally lead to different results.

The objective of this study is to examine and identify the relationship between BMI and mortality from a dynamic prospective. Our analysis is based on the first cohort of the Framingham Heart Study (FHS), which has a long follow-up period and a large number of repeated measures to support our analysis. We first use logistic regression models to demonstrate that different designs on the same population may lead to different results; then we use dynamic survival models to capture the time-varying relationship between BMI and mortality in FHS.

## 2. Materials and Methods

### 2.1. Framingham Heart Study

The Framingham Heart Study (FHS) is an epidemiologic study funded by the National Heart, Lung, and Blood Institute (NHLBI). Multiple cohorts have been recruited into the large study. The original cohort of FHS consists of 5209 men and women between the ages of 30 and 62 from Framingham, Massachusetts. Extensive examinations were carried out every other year on this cohort since 1948 and exam 29 began in April of 2006. In this study, we use information of the first 20 exams (about 40 years of follow-up) of the original cohort for analysis. The exact survival time of each individual is available.

### 2.2. Methods

#### 2.2.1. Logistic Regression Models

We first demonstrate that different study designs shown in Figure 1 can lead to different results using logistic regression models. Three scenarios are created from the FHS: when BMI at a different time point is used (only the location of the bar in Figure 1 changes), when the length of follow-up changes (only the length of the box in Figure 1 changes), when the calendar time of the baseline changes (the bar and the box move simultaneously with calendar time). Within each scenario, a logistic regression model for all-cause mortality is estimated for each design, and the estimated odds ratios (ORs) of BMI are plotted together. We are interested in examining whether there is a consistent changing pattern of estimated ORs within each scenario. The analysis is done separately for men and women since it is likely that there is a gender difference for the relationship between BMI and mortality.

##### (a) When BMI at a different time point is used

The purpose of this analysis is to show whether the result may be different for a study on the same group of subjects when BMI at a different time point is used. We choose the time period from year 30 (exam 16) to year 40 of FHS as the follow-up period. Each measurement of BMI from exam 1 to exam 16 is used to model the mortality within the 10-year period with a logistic regression model. The ORs of BMI of all models within each gender are plotted together against the time difference between the BMI measurement and the baseline of the follow-up period (year 30 of FHS). Only 1,582 (639 men and 943 women) subjects who were alive at exam 16 and had all 16 BMI measurements (exam 1 to exam 16) available are included in the analysis. Subjects excluded are 1762 participants who died before exam 16 and 1865 participants who have at least one missing BMI measurement from exam 1 to exam 16. This strategy is adopted to make sure that all models within each gender are built upon the same group of individuals. The comparison of BMI ORs across all models within each gender demonstrates the effect of using BMI at different time points on the result about the association between BMI and mortality.

##### (b) When the length of follow-up changes

The purpose of this analysis is to show whether the result may be different for a study on the same group of subjects when the follow-up length is different. We set the baseline of follow-up at year 0 (exam 1) and increase the length of follow-up from 6 to 40 years. BMI measured at exam 1 is used for all models. Except 8 subjects with missing BMI measurements at exam 1, 5201 subjects are included in the analysis. There are 18 logistic regression models estimated for each gender. The comparison of BMI ORs across all models within each gender demonstrates the effect of the length of follow-up on the result about the association between BMI and mortality.

##### (c) When the calendar time of the baseline changes

The purpose of this analysis is to determine whether short studies using the traditional design in Figure 1 have a common tendency. We fix the length of follow-up as 6 years and move the baseline (beginning of the follow-up period) from exam 1 to exam 18 to estimate 18 logistic regression models for each gender. For each model, BMI at baseline is used. Sample sizes for all models within each gender are different, varying from 2878 to 687. The comparison of the estimated ORs demonstrates the effect of the calendar time of the baseline on the result about the association between BMI and mortality.

#### 2.2.2. Dynamic Survival Models

The Cox model is a commonly used method in epidemiological studies when survival times are observed. A Cox model has a hazard function in the following form:

(1) $$\lambda (t)\;=\;\lambda_{0}(t)\;\text{exp}\{\text{X}\beta \}.$$

In the model, all covariates are assumed to have fixed effects. This assumption is called the proportional hazards (PH) assumption and it is often violated in reality.

When the PH assumption is violated, that means the association between BMI and the risk of death is not constant but changing within the box in Figure 1. Dynamic survival models can be used to model the change as a function of follow-up time from baseline to the end of the study. A dynamic survival model has a hazard function as:

(2) $$\lambda (t)\;=\;\lambda_{0}(t)\;\text{exp}\{{\text{Z}}_{\gamma }\;+\text{X}\beta (t)\}$$

where ***Z*** denotes all covariates with fixed effects while ***X*** denotes all covariates with time-varying effects. Time-varying coefficients in β(*t*) describe effects (of corresponding covariates) that change with the follow-up time *t* during the entire follow-up period. To estimate such time-varying coefficients, a simple method is to add interaction terms between covariates and the follow-up time *t* into the regular Cox model [13]. Commonly used software such as SAS and STATA can estimate this type of models. Another more flexible approach is time-varying coefficient survival model proposed by Gray [14, 15]. This model uses penalized partial likelihood estimates and can be estimated using the “Coxspline” package of the free statistical software R.

In this part, one model is used to capture the association between BMI and mortality for each gender. Besides gender and age, smoking status (current smoker, yes or no) and systolic blood pressure (SBP, mmhg) are also considered in the analysis. The data set with all this information available is the the original cohort of FHS starting from exam 4 to the end of the 40-year follow-up. The values of BMI, age, smoking status, and SBP are measured at exam 4. The total sample size is 4526 (2005 men and 2521 women).

The Cox model is used at first to examine the relationship between BMI and the risk of death for males and females, separately. The effect of age, blood pressure, and smoking status are controlled. When the test of PH assumption shows significant results, the two different approaches for dynamic survival models are applied to model the associations between BMI and mortality as time-varying functions. All the other variables are allowed to have time-varying coefficients if the PH assumptions for those variables are violated.

With limited data, this paper focuses on showing some evidence that the association between BMI and mortality may change with time rather than modeling the exact changes. For all analyses, BMI and SBP are used as continuous variables and only linear terms are included. The time-varying coefficients survival model shows the change of the linear association between BMI and mortality with time. From the the literature, the association between BMI and mortality is likely to be nonlinear rather than linear. However, a large number of parameters are needed to capture a nonlinear relationship, which leaves limited flexibility for modeling the change of the relationship with time using this small data set. The changes of a linear association show some evidence of the changes of the underlying nonlinear association. This strategy simplifies the analysis and serves the purpose of this preliminary research. Larger data sets and more sophisticated methods will be used for future analysis.

## 3. Results

### 3.1. Logistic Regression Models

The analysis using logistic regression models shows that the result of the association between BMI and mortality may differ when the design of a study changes, such as using BMI measured at a different time or changing the length of the follow-up.

#### 

##### (a) When BMI at a different time point is used

**Figure 2.**
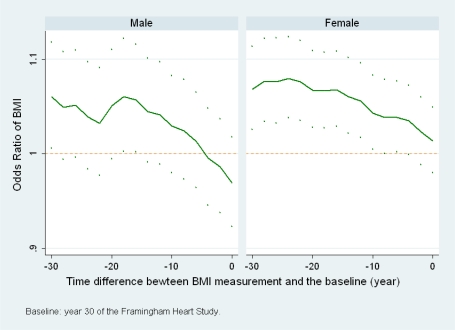
The estimated ORs of BMI for all-cause mortality and their 95% pointwise confidence intervals when measurements made at different time points are used to model the mortality within year 30 to year 40 of the Framingham Heart Study. Death rates: male = 265/639, female = 279/943. Time 0 denotes year 30 of FHS, the beginning of the follow-up period.

Each measurement of BMI at exam 1 to exam 16 is used to model the mortality in year 30 to year 40 of FHS.Figure 2 presents the estimated ORs (connected lines) of BMI and their point-wise 95% confidence intervals (dots). The horizontal axis denotes the time difference between BMI measurement and baseline, the beginning of the 10-year period. A time difference of −30 years means BMI is measured 30 years before the baseline. Age effect on mortality is controlled by including age at exam 16 (year 30) as a covariate. For each gender, the same group of individuals are included in all of the 16 models and the only difference among these models is the time when BMI is measured.

Both plots in Figure 2 show overall downward trends. For women, the ORs for BMI measured more than 10 years earlier than the baseline are significantly greater than 1 (95% confidence intervals are above 1), while those for BMI measured within 10 years are not. For men, although most ORs are not significantly different from 1, the decreasing trend is more obvious than that of women and the ORs for BMI measured within 6 years to the baseline are even less than 1. Since the only difference among all the models within each gender is the time when BMI is measured, Figure 2 suggests that a study may get a different result if BMI measured at a different time point is used. Here, BMI measured earlier means BMI at younger ages for these individuals. What Figure 2 suggests is that using BMI at younger ages instead of at the baseline of a study may obtain a different result. For women, using BMI at younger ages may show stronger direct linear associations. For men, when an inverse linear association is observed using baseline BMI, using BMI at younger ages may show an opposite result of direct linear association.

##### (b) When the length of follow-up changes

**Figure 3.**
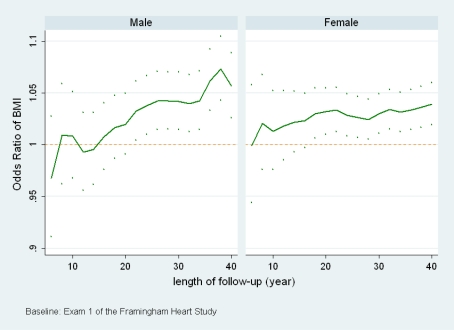
The estimated odds ratios of BMI measured at exam 1 and their 95% pointwise confidence intervals for all-cause mortality in different follow-up periods. Sample size: 2333 men and 2868 women.

Figure 3 shows the ORs of BMI measured at exam 1 to model the mortalities of men and women within 6 years to 40 years after exam 1. There are 18 models in total for each gender. The age effect is also controlled in all the models using age at exam 1. The same group of individuals are included in all models for each gender and the only difference among the 18 models of each gender is the length of follow-up.

Figure 3 shows overall increasing trends of the ORs for both men and women. For men, the OR is less than 1 when the length of follow-up is the shortest (6 years), then it increases consistently as the length of follow-up increases. The same pattern is observed for women except that the change is less obvious. This analysis suggests that the direct linear association between BMI and mortality may be stronger if a study has a longer follow-up period. Also, the change for men may be more obvious than that for women. For men, an inverse linear association may be observed when the length of follow-up is short.

##### (c) When the calendar time of the baseline changes

The analysis in part (a) suggests that the more recent BMI is used, the more likely it is to obtain no linear association for women or an inverse association for men. The analysis in part (b) suggests that the shorter the length of follow-up, the more likely it is to obtain the same result. We design the third scenario to examine whether short studies using baseline BMI tend to obtain similar results.

**Figure 4.**
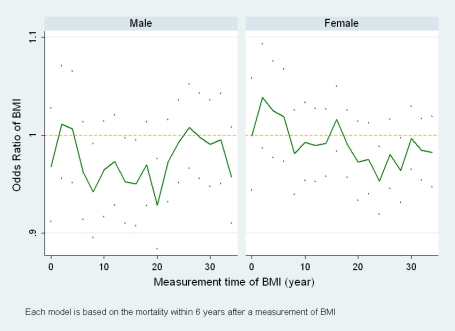
The estimated odds ratios of BMI for all-cause mortality and their 95% pointwise confidence intervals when short follow-up periods and baseline (the beginning of each follow-up period) measurements of BMI are used.

Figure 4 shows the ORs of BMI for 18 models of each gender. Each model uses BMI and age at one of the first 18 exams as covariates and 6 years after the exam as the follow-up period. In the figure, 14 odds ratios for men and 15 odds ratios for women are less than 1. Compared with those of men, most odds ratios for women are close to 1. For example, 11 out of the 18 odds ratios are no more than 0.02 different from 1 for women. None of the ORs is significantly greater than 1 while 4 ORs for men and 2 ORs for women are significantly lower than 1 (95% confidence intervals are below 1).

With no obvious changing patterns in either plot of Figure 4, this result suggests that a short study using baseline BMI to examine the association between BMI and mortality based on FHS tends to conclude with no linear association for women and an inverse association for men. Since the change of the baseline also means a study on a population at different ages, Figure 4 suggests that the tendency for short studies to conclude with no linear association or inverse associations may be true for the FHS population at both young and old ages.

### 3.2. Dynamic Survival Models

First a Cox model is used to model the association between BMI and mortality with age, smoking status, and blood pressure controlled. We tested the proportional hazards (PH) assumptions for each covariate in the models. The test results using STATA 10 are in Table 1.

In Table 1, the linear effect of BMI in the Cox model for men is insignificant (*p* = 0.876), but the test of PH assumption is highly significant (*p* = 0.0001). The tests of the coefficients in the Cox model are Wald tests and the test of PH assumption used in STATA 10 was proposed by Grambsch and Therneau [16]. This result suggests that the hazards ratio of BMI changes significantly with time. The tests of the PH assumptions for age, SBP, and smoking status are all significant. As for women, the test of BMI effect in the Cox model is significant (*p* = 0.011) and the test of PH assumption of BMI is a borderline case (*p* = 0.060). The tests of the PH assumptions of age and SBP for women are also significant while that of smoking status is not significant.

**Figure 5.**
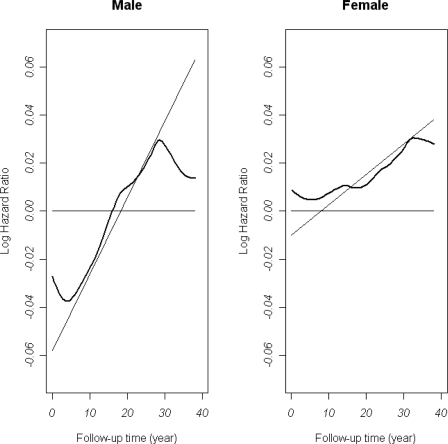
The estimated time-varying log hazard ratio functions of BMI. 1) thin lines: Cox model with interactions with time. 2) thick curves: Time-varying coefficient survival models. The effects of age, SBP, and smoking status are controlled.

Figure 5 shows the estimated time-varying log hazard ratios of BMI for men and women separately using the two methods described in the method session. The straight lines are estimates from the Cox models with linear interaction terms using software STATA 10. The estimated function for men is −0.58 + 0.0032*t* and that for women is −0.01 + 0.0013*t*, where t is the follow-up time (years). The smooth curves in Figure 5 are the estimates of the time-varying coefficient survival models using software R. We used quadratic splines and 10 knots for each model. All the hazard ratios of other covariates with the PH assumptions violated are allowed to change with time (not shown). From the figure, we can see that the estimated log hazards ratios using these two methods have similar increasing patterns, especially for women. Overall, the hazards ratios of BMI increase over time for both men and women, and the increase for men is more obvious than that for women. In the last 10 years of follow-up, the hazards ratio for men based on the flexible model decreases, which can not be captured by the Cox model with linear interactions. The estimated hazards ratio of BMI for men is less than 1 (log hazards ratio in Figure 5 is less than 0) during the first 15 years of the follow-up period and is greater than 1 afterwards. The hazards ratio for women is close to 1 (log hazards ratio in Figure 5 is close to 0) then gradually increases with the follow-up time.

The results using dynamic survival models clearly show the changing pattern of the association between BMI and mortality with the follow-up time, especially for men.

## 4. Discussions

The purpose of our analyses is to examine and identify some evidence that the association between BMI and mortality may not be static so that studies with different designs will capture different pictures of the association of BMI and mortality. We demonstrated, using a single data set, that the relationship between BMI and mortality is, for this data, a complex dynamic relationship where the results obtained depend heavily on the designs of the study and that the relationship does not appear to satisfy the proportional hazards assumption. We also observed from this study that the dynamic feature is stronger for men than for women. However, the same analysis may not be repeatable in other studies if they do have have a large number of repeated measures of BMI within a long follow-up period. Our conclusion is limited to this particular study and population.

There are some findings in the literature that are consistent with our results from different aspects. Some published studies include all individuals alive at baseline for analysis, while others excluded deaths within the first several years of follow-up from analysis [17–20]. This strategy of dropping early deaths sets the real follow-up period to start several years after the time when BMI is measured. Our analysis suggests that this strategy will lead to overall stronger direct associations between BMI and mortality, especially for men (as in Figure 2). Allison *et al*. performed a meta analysis about the effect of dropping earlier deaths (in 4–6 years) and found that “the effect of excluding subjects who died early was greater for men than that for women” [21]. According to Figure 2, our analysis also suggests that this changing relationship may last well beyond 4–6 years. One study in the literature proposed the average-adulthood BMI rather than baseline BMI [22]. Another study found that dropping early deaths affects the analysis using BMI at baseline (average age at baseline is 73) but do not affect the analysis when BMI at age 21 is used [20]. All these findings in the literature showed that using BMI measured at different time points or changing the follow-up period (drop early deaths) may affect the analysis.

Our analyses are limited in another way in that only the linear association between BMI and mortality is under consideration. Our analysis does not show directly, but suggests the change of the shape of the association between BMI and mortality. In scenario b) with fixed BMI and changing length of follow-up (Figure 3), because the individuals and BMI measurement are the same for all models within each gender, the observed changes of the linear association imply the change of the shape of the underlying association between BMI and mortality. A non-linear relationship, if one exists, would show a much more complex dynamic pattern than the pattern shown in this study. Our future research is to model how an unspecified nonlinear association changes with time and interpret the changes. Also, more research will need to be done with other data to verify the dynamic feature observed in FHS.

To our knowledge, despite its limitations, our study is the first study to examine the association of BMI and mortality from a dynamic prospective that it provides a unified view of the impact of the study design on the result. If different designs lead to different results, then which design better capture the causal effect of body weight on mortality? For an observational study about body weight and mortality, researchers may consider obtaining BMI at multiple time points to compare the differences, having a relatively longer follow-up period, and applying appropriate alternative models when the proportional hazards assumption is violated.

## Figures and Tables

**Table 1.** Cox models and the tests of the proportional hazards assumptions for the Framingham Heart Study.

Analysis Result for Men			
Variables	Hazards Ratio	p-value	Test of PH assumption (p-value)

Age (5 years)	1.57	< 0.001	0.034
sbp (10 mmHg)	1.15	< 0.001	0.026
Smoking	1.42	< 0.001	< 0.0001
BMI	1.00	0.876	0.0001

Analysis Result for Women			
Variables	Hazards Ratio	p-value	Test of PH assumption (p-value)

Age (5 years)	1.58	< 0.001	0.001
sbp (10 mmHg)	1.12	< 0.001	0.017
Smoking	1.42	< 0.001	0.570
BMI	1.02	0.011	0.060
